# Deep learning: survey of environmental and camera impacts on internet of things images

**DOI:** 10.1007/s10462-023-10405-7

**Published:** 2023-02-06

**Authors:** Roopdeep Kaur, Gour Karmakar, Feng Xia, Muhammad Imran

**Affiliations:** 1 Institute of Innovation, Science and Sustainability, Federation University Australia, Ballarat, VIC 3353 Australia; 2 School of Computing Technologies, RMIT University, Melbourne, VIC 3000 Australia

**Keywords:** Camera lens distortions, Deep learning, Environmental impacts, Internet of things, Image quality

## Abstract

Internet of Things (IoT) images are captivating growing attention because of their wide range of applications which requires visual analysis to drive automation. However, IoT images are predominantly captured from outdoor environments and thus are inherently impacted by the camera and environmental parameters which can adversely affect corresponding applications. Deep Learning (DL) has been widely adopted in the field of image processing and computer vision and can reduce the impact of these parameters on IoT images. Albeit, there are many DL-based techniques available in the current literature for analyzing and reducing the environmental and camera impacts on IoT images. However, to the best of our knowledge, no survey paper presents state-of-the-art DL-based approaches for this purpose. Motivated by this, for the first time, we present a Systematic Literature Review (SLR) of existing DL techniques available for analyzing and reducing environmental and camera lens impacts on IoT images. As part of this SLR, firstly, we reiterate and highlight the significance of IoT images in their respective applications. Secondly, we describe the DL techniques employed for assessing the environmental and camera lens distortion impacts on IoT images. Thirdly, we illustrate how DL can be effective in reducing the impact of environmental and camera lens distortion in IoT images. Finally, along with the critical reflection on the advantages and limitations of the techniques, we also present ways to address the research challenges of existing techniques and identify some further researches to advance the relevant research areas.

## Introduction

Recently, a large volume of data is being generated by the IoT (Gubbi et al. 2013) devices. The analysis of such big data is a cumbersome process. Therefore, an advanced machine learning technique called DL (LeCun et al. 2015) is required to extract the value from these IoT data. DL techniques have been widely used in computer vision, especially in difficulties of visual classification in which the latest algorithms perform better than humans. Various works have been done in which Deep Neural Networks (DNN) (Yi et al. 2016) are used to classify high-resolution images into different categories (Krizhevsky et al. 2012; Simonyan and Zisserman 2014; Lee et al. 2015). Therefore, DL algorithms are in great demand in image processing. There are various cloud-based services by Google, Amazon, and Microsoft in which users do not need to train their models for image processing.

Recently, Google made Cloud Vision Application Programming Interface (API) (Ofoeda et al. 2019) for the analysis of images. However, (Hosseini et al. 2017) demonstrated that Google’s API is not robust to the noise in images. Google API can detect objects and faces and also read out words in images. Nevertheless, when noise is added to images, the same API produces different outputs. If the noise from noisy input images is filtered, it results in the same output as the original images. Therefore, the efficiency of DL algorithms can be enhanced by pre-filtering noisy input images.

A DNN, namely Alexnet (Lu et al. 2019) has also achieved good performance in image processing. However, in practical applications, various kinds of impacts occur during image capturing. These impacts can be weather conditions, lighting conditions, or camera parameter impacts. Weather condition parameters include rain, snow, fog, wind, shadow, and darkness (Kapoor et al. 2016), while camera parameters are lens blurriness, dirtiness, and distortions (e.g., barrel distortion) (Buades et al. 2005).

Research shows these impacts can affect the performance of DL algorithms (Temel et al. 2019, 2017). Consequently, it is necessary to consider the impact of these parameters while using DL techniques for image processing. For this, there exist some DL-based techniques to analyse and reduce both environmental (Kazerouni et al. 2019; Tschentscher et al. 2017) and camera impacts (Lu et al. 2017; Liu et al. 2018) on IoT images. The existence of these techniques necessitates presenting their state-of-the-art approaches in a survey paper. So that the research community can perceive the impacts and address them accurately. In this paper, for the first time, we present an SLR (Kitchenham et al. 2009) on DL-based techniques that are used to analyse and reduce the impacts of environmental and camera parameters on IoT images.

The contributions of the paper are summarised as follows:

1. We emphasise the significance of IoT images and how they are used in their respective applications.
2. We compile state-of-the-art DL-based approaches for the analysis of the impacts of dynamic outdoor environments and camera lenses on the quality of IoT images.
3. We incorporate the latest works on DL-based techniques for the reduction of both environmental and camera lens impacts on image quality.
4. We present some further research to advance the impact analysis and reduction techniques for IoT images.

The structure of the paper is organised as follows: the review methodology is described in Sect. 2. Section 3 emphasises the applications of IoT images in various application domains. In Sect. 4, we provide diverse DL techniques for the analysis of impacts on IoT images. The reduction of environmental and camera parameter impacts on IoT images is demonstrated in Sect. 5. Section 6 brings further research challenges. Finally, the conclusion is given in Sect. 7.

## Review methodology

The SLR has recently been used to conduct the literature survey. SLRs are logical and systematic methods of identifying, evaluating, and interpreting existing approaches related to a particular research problem. As it lays the foundation for primary research, SLR can reduce the possibility of bias and is regarded as secondary research (Keele 2007). Therefore, in this paper, we opted to present the current state-of-the-art of environmental and camera impacts on DL, which is the main purpose of this survey.

The following research questions have been established to achieve the above objective:

Research Question-1 (RQ-1): What is the significance of the analysis of IoT images?

Research Question-2 (RQ-2): What are the different DL techniques used for the analysis of various environmental and camera impacts on IoT images?

Research Question-3 (RQ-3): Which DL techniques are being used to reduce the environmental and camera effects on IoT images?

Note that considering the purpose and scope of the study, we have selected above-mentioned three questions that emphasise the significance, motivation and contributions of the study. To answer the research questions outlined above, appropriate keywords, research databases, and include and exclude criteria were selected. As a final search string, we used the following search keywords:“IoT images”,“IoT image capturing”, “IoT applications”, “environmental impacts”, “camera impacts”, “machine learning”, and “deep learning”. We also combined these keywords to get better results. Research databases such as Science Direct, IEEE Xplore, Google Scholar, ACM, and Springer, as well as websites such as www.statista.com and www.towardsdatascience.com were utilised to search for relevant topics.

The following criteria for include and exclude was utilised:

Inclusion criteria are (i) English-language data sources, (ii) articles and papers containing searched keywords in their text, and (iii) peer-reviewed academic sources, such as conference proceedings, journal articles, and book chapters.

In our search criteria we excluded (i) data sources written in languages other than English, (ii) articles mentioning the search keywords only in the references, and (iii) resources published before 2002.

Search results: about 19,100 search results were returned from Google Scholar that were relevant to the keywords. We apply the filtering criterion of “since 2000". Further, three main areas (i) application of IoT images (ii) analysis of environmental and camera impacts (iii) reduction of environmental and camera impacts are focused.

**Fig. 1:**
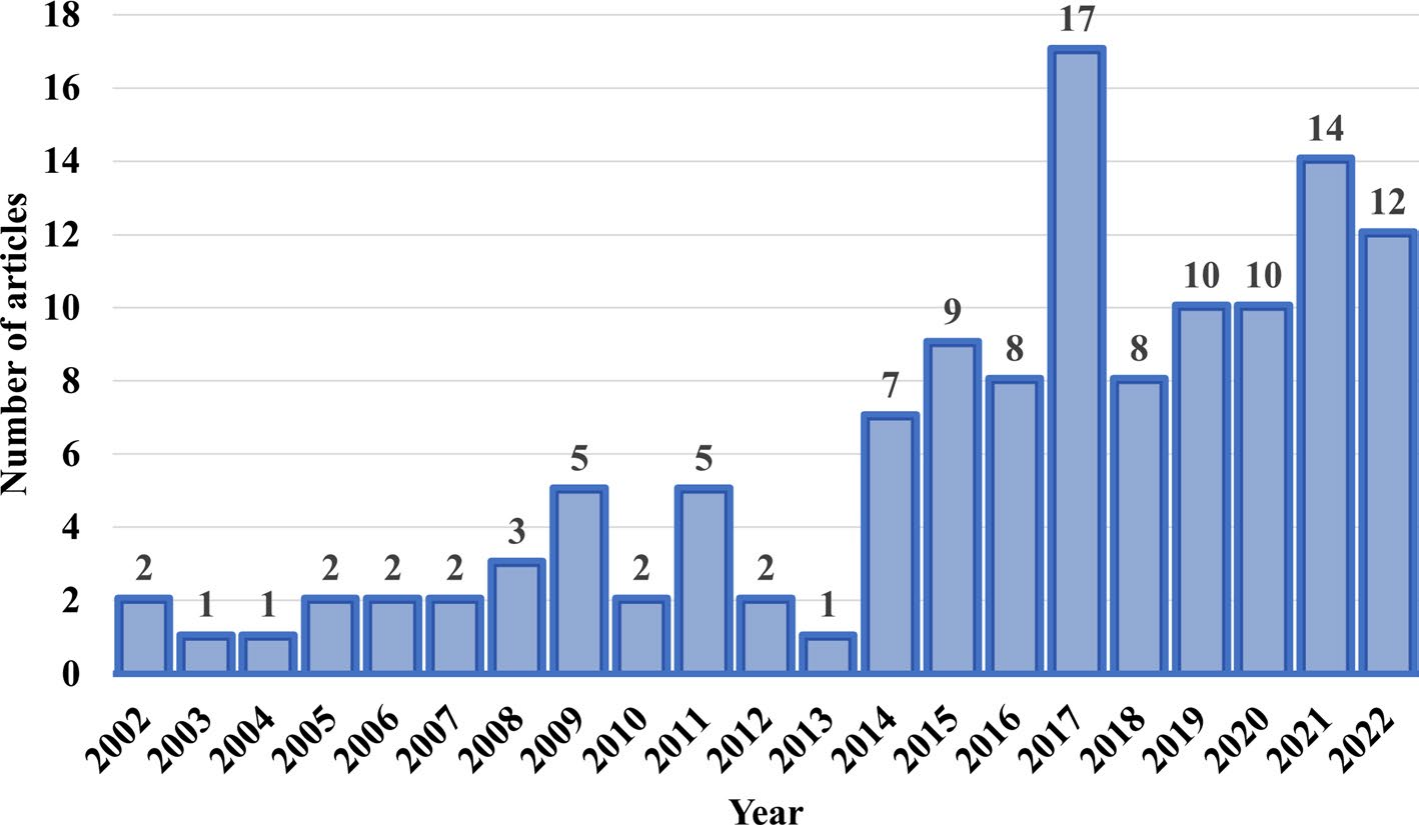
Annual publications in IoT and various DL techniques used for the analysis and reduction of environmental and camera lens distortion impacts

**Fig. 2:**
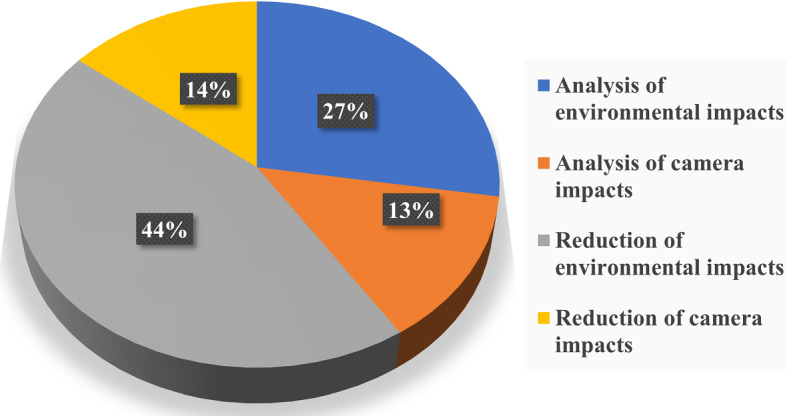
Percentage of articles published in analysis and reduction of environmental and camera impacts

The 123 papers are arranged according to the annual publications in IoT and various DL techniques used for the analysis and reduction of environmental and camera lens distortion impacts. The distribution of articles is clearly represented in Fig. 1. The utilisation of DL techniques for environmental and camera impact analysis and reduction was restricted in the first decade of the 21st century because the third wave of DL emerged in 2006 with a breakthrough (Bansal 2020). It has rapidly increased from 2014 onwards and was maximum (of 17 articles) in 2017. Further consideration was given to articles that mention the impacts of environmental and camera parameters on DL algorithms which is the principal objective of this article. This will enlighten us about the emergence and effectiveness of various DL techniques for the analysis and reduction of environmental and camera impacts. There are 36 primary articles that use DL taken from 123 papers which are shown in Fig. 1 and included in the main body of this review. The distribution of these 36 articles among analysis and reduction of environmental and camera impacts is demonstrated in Fig. 2.

There are two major groups of selected primary articles representing the analysis of environmental and camera impacts and the reduction of environmental and camera impacts. According to the search results, there is more number of articles on the analysis and reduction of environmental impacts compared with the camera impacts. Most research is happening on the reduction of environmental impacts which is 44%. The second-highest number (27%) of articles is on the analysis of environmental impacts. Figure 2 also shows there has been a substantial amount of research i.e., 14% and 13% on analysis and reduction of camera impacts. Active research is going on for the reduction of environmental impacts and its popularity is increasing over the years. It shows the ever-increasing demand for DL algorithms in the analysis and reduction of environmental impacts. However, on contrary, still, there is a need to explore more DL techniques for the analysis and reduction of camera impacts.

The research questions mentioned above are explained in the following sections. RQ-2 is well elaborated through Sect. 4. In Sect. 5, RQ-3 is covered in detail. RQ-1 is addressed in the following section.

## Applications of IoT images

**Fig. 3:**
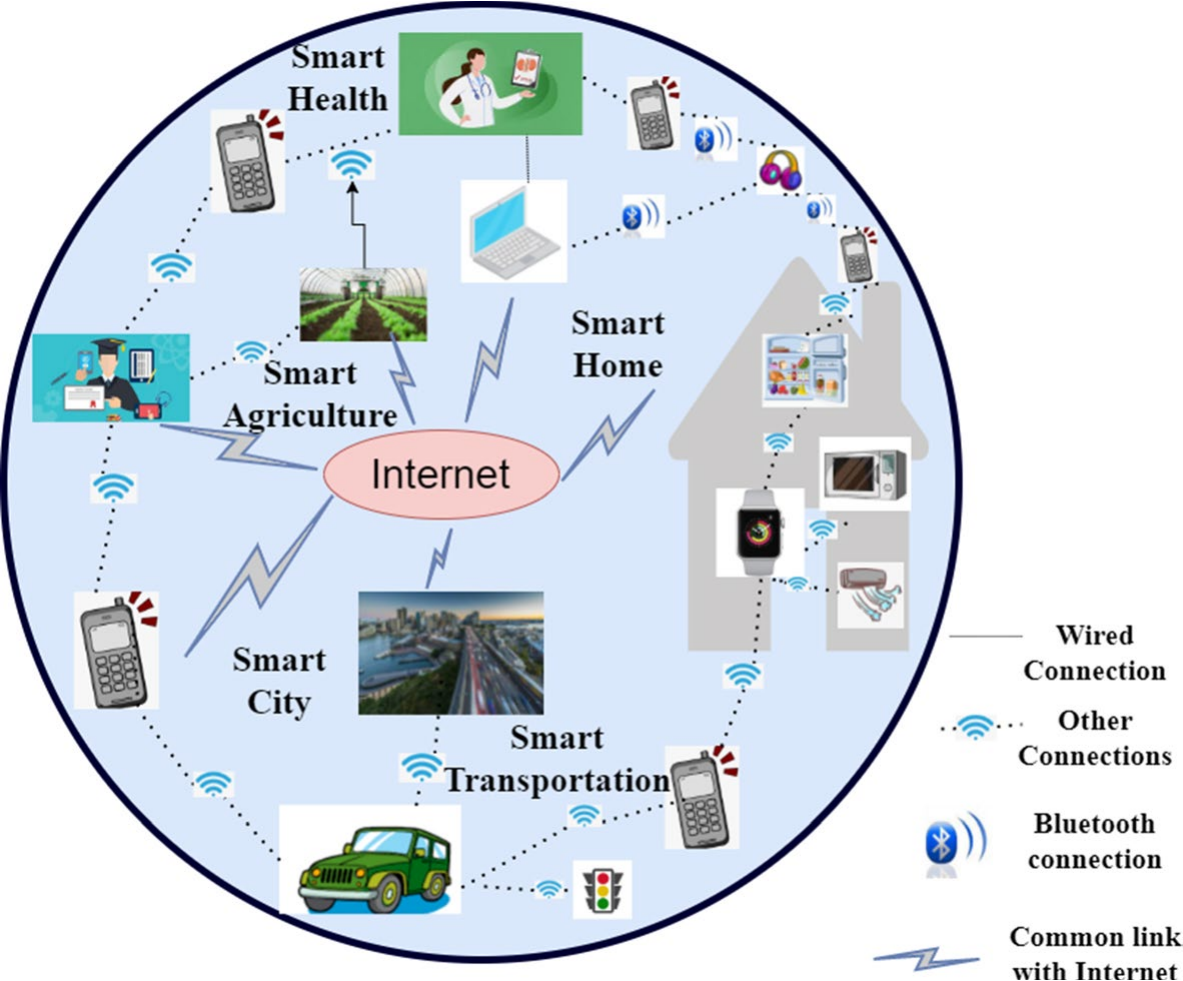
IoT. Note, other connections indicate Wi-Fi/cellular communications/IoT network connections

The concept of the IoT came from a United States research scientist, Peter T. Lewis, in 1985 (Sharma 2016). IoT is regarded as the future of the Internet because the backbone of IoT is the Internet. The vision of IoT is to connect all smart devices (either things or humans carrying/using those smart devices) with the Internet. Figure 3 shows an example of IoT where everything (e.g., city, field, home, hospitals, vehicles) is connected to the Internet. Wireless technologies, such as 2 G/3 G/4 G, WiFi, blacktooth, etc., have been used in heterogeneous IoT applications, where billions of devices are connected by wireless communication technologies. 2 G networks (currently covering 90% of the world’s population) are for voice, 3 G (currently covering 65%) is for data and voice, and 4 G is for broadband internet experiences (since 2012). In recent years, 4 G technology has significantly enhanced the capability of cellular networks to provide IoT devices with Internet access (Akpakwu et al. 2017). In 2012, Long-Term Evolution (LTE) (Ghosh et al. 2010) emerged as the fastest and most consistent type of 4 G compared to other technologies, such as Worldwide Interoperability for Microwave Access (WiMax) (Taylor 2011), ZigBee (Nokia 2017), SigFox (SigFox 2018), Long Range (LoRa) (Vangelista et al. 2015), etc. The 5 G networks and standards are expected to solve challenges facing 4 G networks, such as more complex communication, computational capability, intelligence, etc, to meet the needs in industry 4.0 (Zhang and Chen 2020) and smart environments (Costanzo and Masotti 2017), (Li et al. 2018). Next-generation mobile communication and network technology is 6 G. The development of the 6 G mobile communication system (after the previous five generations from 1 G to 5 G) will be a revolution in mobile communication. 6 G will be a holographic, multidimensional network with the ability to expand coverage over air, sea, and land, and integrate with AI, IoT, and blockchain (Lu and Ning 2020). AI, 5 G/6 G, and Quantum Computing are rising technologies that will drive innovations and contribute significantly to the future development of Industry 4.0 that enables moving industries to a higher level. The new technology is capable of integrating both new and classical Industrial 4.0. Future industries and technologies will be transformed by artificial intelligence, 6 G, and quantum computing (Sigov et al. 2022).

IoT devices are considered smart because they are assumed to have human-like capabilities. These capabilities include intelligent processing including inferencing for decision making and two ways of interacting with other smart devices. These capabilities also incorporates data transmission, joining a group for a particular mission and removing from that group after mission completion. For this reason, these devices have processors, transmitters, memories, Global Positioning System (GPS) (El-Rabbany 2002), Radio-frequency identification (RFID) (Landt 2005) and the relevant software and tools that can enable these devices to do intelligent processing.

**Fig. 4:**
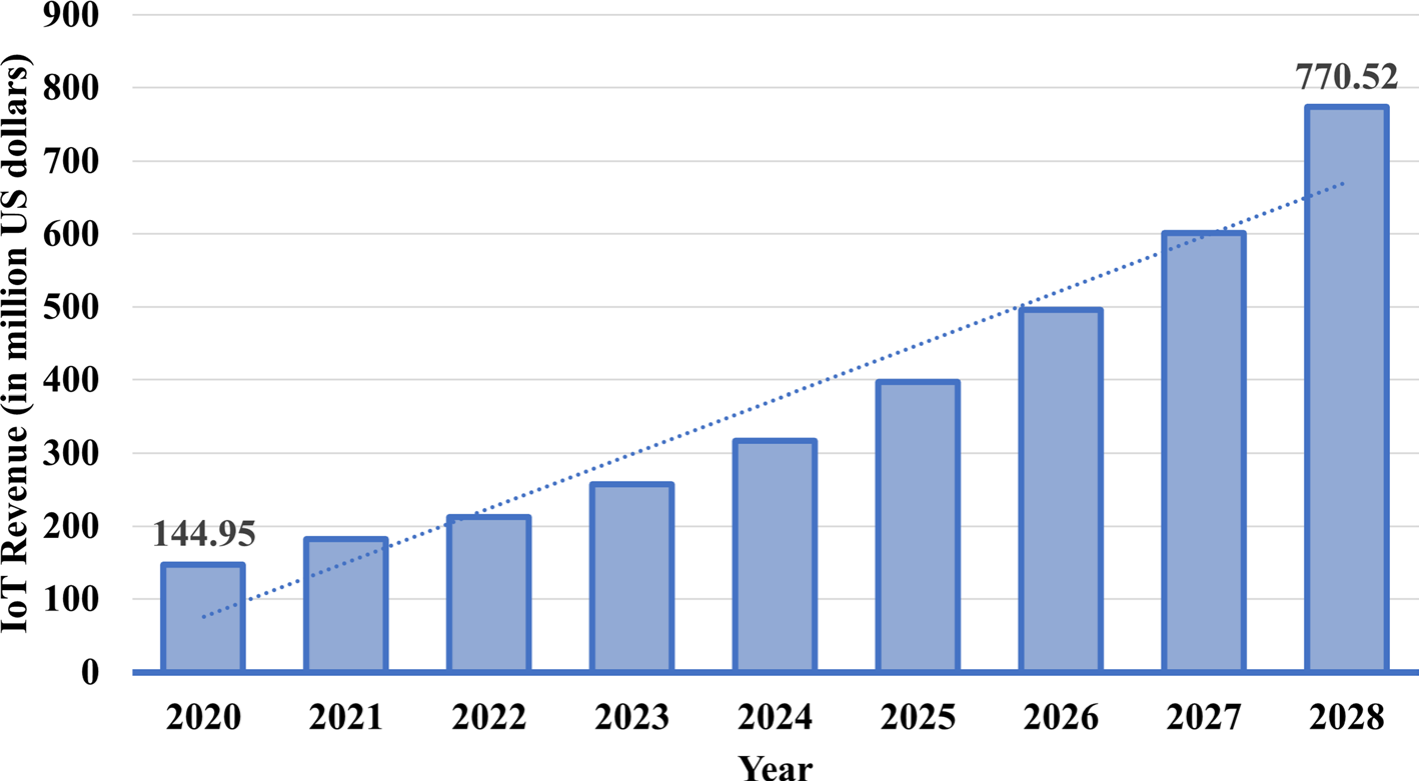
Global IoT revenue from 2020 to 2030 (in billion US dollars) (Som and Kayal 2022)

**Fig. 5:**
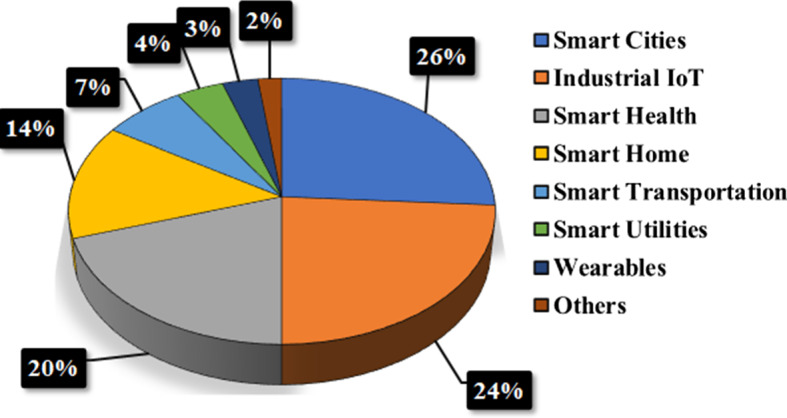
IoT utilisation in various application domains

IoT revenue worldwide is ascending exponentially from the year 2020 and will increase in the future as shown in Fig. 4. Various sources suggests that it will be more than 1.5 Trillion United States Dollar (USD) by 2030 [36], (Morrish and Hatton 2020; Biswal 2021). For example, in 2020, IoT revenue was USD 144.95 million, but by 2028, it will increase to USD 770.52 million (Som and Kayal 2022). Currently, IoT is burgeoning in most application domains like industry (Javed et al. 2018) such as digital industry, robots, smart grid, and energy. It is also highly utilised in applications such as smart cities (Zhu et al. 2015), smart health (Islam et al. 2015), smart home (Perera et al. 2014), smart transportation (Da Xu et al. 2014), smart agriculture (Talavera et al. 2017) and others (refer to Fig. 5). Figure 5 shows the most IoT usage is in smart cities (26%), smart industry (24%), smart health (20%), and smart homes (14%). We expect smart transportation, smart utilities, and smart health (wearables) will increase the adoption of IoT technologies in the future. IoT technology may facilitate intelligent transportation and further enhance greenness and sustainability (Golpîra et al. 2021). Examples of such IoT applications are presented in Fig. 3. In particular, for smart homes, as shown in Fig. 3, mobile phones, refrigerators, Air Conditioner and microwaves are connected to smart watches through Wireless-Fidelity (Wi-Fi), cellular communications or IoT network connections (these connections shown in Fig. 3 are referred to as other connections). Similarly, in smart health, healthcare service providers including hospitals and clinics are connected to peripheral devices (for example, blood pressure monitors, heart rate monitors, webcams, headphones), research departments, and other handheld devices through wired, blacktooth, or other connections as depicted in Fig. 3. Recently, the Severe Acute Respiratory Syndrome Coronavirus 2 (SARS-CoV-2) (Pal et al. 2020) is influencing different aspects of life and industry. IoT plays a crucial role in this disease as an IoT-based system is able to manage and balance the impact of SARS-CoV-2 in smart cities by cluster identification which identifies people who are not wearing face masks (Herath et al. 2021). Other examples include monitoring vaccine temperature using IoT (Almars et al. 2022).

**Table 1:** Contemporary IoT applications

Year	Purpose	Application domain	Data type	IoT N/W	ICT platform	IoT devices	Impact (type)
2019 (Li et al. 2019)	An efficient system with less complexity and energy requirement image communication system for IoT based monitoring applications	Smart agriculture and transportation	Image	Wi-Fi	Cloud based	Sensors	Image transmission insecurity
2021 (Piccialli et al. 2021)	An IoT based system which can predict the parking occupancy in the streets and enhance the flow of cars in the overcrowded areas	Smart transportation and city	Numerical	LPWAN	Non-cloud based	Sensors	Air pollution and noise traffic
2021 (Mabrouki et al. 2021)	Weather (temperature, humidity) monitoring system for remote places with Arduino based wireless sensor networks	Smart environment	Numerical	Wi-Fi	Non-cloud based	Temperature, humidity, and CO2 sensors	Pollutants
2021 (Sahu et al. 2021)	Proposed a security mechanism for e-healthcare applications	Smart health	Graphical images	Wi-Fi	Cloud based	Heart rate sensors	Insecurity
2021 (Xie et al. 2021)	Environment monitoring system in shallow sea	Smart environment	Numerical	NB-IoT	Cloud based	Environment monitoring sensors	Unreliability
2021 (Savic et al. 2021)	Demonstration of anomaly detection for cellular IoT networks which can be utlilized in smart logistics through the real world data study	Smart logistics	Numerical	NB-IoT	Non-cloud based	CIoT (Cellular IoT) user equipment	Anomoly detection

Since IoT contains sensor networks and thus has sensing capability, image processing becomes an indispensable part for many IoT applications. For automation, understanding circumstances around the application-focused things (e.g., application-specific scenarios, events) and dealing with the uncertainty associated with applications, image processing has been essential for IoT-based smart applications. For this reason, like other data types such as numeric and textual data, IoT images are being applied in various smart applications. Some contemporary IoT applications are provided in Table 1. Table 1 includes applications associated with the most application domains (e.g., smart agriculture, smart health, smart transportation) shown in Fig. 5.

Agriculture is one of the sectors in which the combination of IoT and image processing lead to efficient and cost-effective methods. These methods increase production to a great extent. IoT images can be utilised in smart agriculture to predict crop yield and to detect various crop diseases. Temperature, humidity, and soil moisture content can be predicted using the combination of IoT sensing networks and image processing networks. Kapoor et al. (2016) presented an approach that determines different environmental (under different sunlight conditions) and man-made factors (pesticides, insecticides) which could impact the production of agriculture. In this approach, different sensors and image-capturing cameras are assembled on an Arduino and the data are captured from these sensors. The camera is stored in an Secure Digital (SD) card and then further processing is done with algorithms in MATLAB. A database is created for the histogram analysis. Four sets of the same plant are taken. A healthy set of plants is kept in set l. The plants in set 2 are kept in extreme sunlight under moist conditions. Low to moderate sunlight is provided to set 3 with little to no water. The fourth set is treated with excesses of *N*, *P*, and *K* fertilizer under normal environmental conditions. There were visible morphological differences between each of these sets. As a result of these visible morphological differences, each plant can be analysed through the use of histograms. Depending on the set, each histogram plot will be different. Thus, the algorithm analyses the given image and identifies the specific problem that affected the given plant. Therefore, information collected concerning temperature, humidity, and moisture can be used to predict the health of the plant.

Not only in agriculture, but IoT images are also an indispensable part of smart health. The combination of IoT images and other networks give us the option of home-based healthcare and telehealth services. The detection of diseases is easily possible through these services while the old patients being at home. For example, Liu et al. (2019) demonstrated an approach to perform the acquisition and processing of teeth images with the combination of IoT networks and DL techniques. The approach decreases the mean diagnosis time by 37.5% which increases the number of patients who needs to be treated by 18.4%.

Moreover, IoT images are also beneficial for smart transportation as mentioned in Table 1 in which the early prediction of parking spaces is done and linked to a mobile app Users can know about vacant parking spaces in congested areas through that mobile app It results in less consumption of fuel and saves time as well. Similar to the parking prediction, a traffic prediction technique was introduced by Goel et al. (2017) in which authors presented a smart traffic monitoring system with Closed-Circuit Television (CCTV) (Cieszynski 2006) cameras. Based on the traffic, an alert is given to the people about highly congested roads and alternate paths are suggested to them. Likewise in smart transportation and smart health, IoT images are also utilised in wildlife or forest protection. These images are used to classify the animals through IoT image networks and DL models so that we can save endangered species of animals and plants.

IoT images are also an indispensable part of the smart industry which enhances the manufacturing of the product and thus increases overall production. Hsu et al. (2017) proposed a system for the automatic production of steel products.

Beyond these above applications, IoT images can also be used in smart cities, smart homes, transportation, and logistics. Hence, to apply IoT image networks efficiently for the above-mentioned applications, there is a need for good quality and reliable images. Since the efficacy of IoT image-based applications is heavily affected by the image quality, it is of utmost crucial to make sure that good quality images are collected through the IoT networks.

Because lighting, time, the position of the camera, and weather conditions (e.g. rain, wind, snow, fog) are all factors that contribute to capturing images in an outdoor environment. In windy weather, for instance, objects may lose their clarity. This results in blurry images. Other important factors include the time, camera setting, and distance at which images are taken in uncontrolled conditions. There are also differences in the images taken in the morning, afternoon, and at night, which are caused by differences in lighting conditions (Kapoor et al. 2016), light sources, and the number of shadows on the image which impacts the processing accuracy. As a result, outdoor images require consideration of all the above-mentioned effects in the analysis (Fathi Kazerouni et al. 2019). Without considering all possible outdoor environmental impacts, decisions derived from outdoor image analysis can be erroneous. Therefore, many techniques are available to analyse the environmental and camera impacts on images over the decades. In these techniques, the quality of images is objectively assessed by many image quality assessment metrics such as Peak Signal-to-Noise Ratio (PSNR) [60], Mean Square Error (MSE) (Aziz et al. 2015), Oriented FAST and Rotated BRIEF (ORB) (Rublee et al. 2011) and Structural Similarity Index (SSIM) (Z Wang et al. 2004; Kaur et al. 2022). Research also shows the prominent evolution and ever-increased adoption of DL applications to analyse the environmental and camera impacts on IoT images. Consequently, various DL-based techniques for the environmental and camera impact analysis on IoT images are described in the following section which also answers our RQ-2.

## DL techniques used for analysing the impacts on IoT images

There are a variety of DL techniques (depicted in Table 2) that are used for analysing the impacts on IoT images. Table 2 summarises the purpose, research challenges, impact type, DL techniques, and dataset used for each approach. For example, Table 2 shows Zhou et al. (Zhou et al. 2017) analysed the impact of lens blurriness using DNN classifiers (LeNet-5, MatConvNet) and MNIST, CIFAR-10 and ImageNet datasets. Some approaches to alleviate those impacts are also articulated in this approach. These impacts can be categorised into two major classes: (i) environmental and (ii) camera lens distortions.

**Table 2:** Different DL techniques for the analysis and reduction of impacts due to environmental and camera parameters while image capturing

Approach/Year	Purpose	Research challenge addressed	Type of impact	DL type	Dataset used
To study the performance of different DNN classifiers, 2016 (Dodge and Karam 2016)	Examine the impact of different types of image distortion such as JPEG compression, noise on the classification of images on DNN	The impact of image distortion on different four neural networks is analysed and Visual Geometry Group-16 (VGG-16) (Alippi et al. 2018) network outperforms for various distortions	Contrast, JPEG compression, noise, blur (camera and environmental impact)	Caffe Reference, VGG-CNN-S, VGG-16, GoogleNet	ImageNet (Non-synthetic) (Russakovsky et al. 2015)
To enhance the Google cloud robustness, 2017(Hosseini et al. 2017)	Improve Google Cloud Vision API’s robustness to noise	Improvement of system robustness to noise by adding noise filter	Gaussian noise (environmental impact)	Google’s cloud vision API (DNN)	ImageNet (Deng et al. 2009), Faces94 [70] (Both)
Testing of DNN efficiency, 2017 (Lu et al. 2017)	Check the effectiveness of DNN with varying distance of camera and angle	Improvement in DNN for the classification of images under the impact of camera distance and angle	Distance and angle (camera impact)	YOLO detector (DNN)	MSCOCO (Lin et al. 2014), German traffic signs dataset (Stallkamp et al. 2011) (Synthetic)
To check the efficacy of DNN classifiers, 2017 (Zhou et al. 2017)	Analyse the impact of blurriness and noise on DNN classifiers	Provided approaches to reduce the effects of image distortion	Blurriness (camera impact) and noise (environmental impact)	LeNet-5, MatConvNet (Deep Convolutional Neural Network (DCNN))	MNIST, CIFAR-lO (Krizhevsky et al. 2009), ImageNet (Deng et al. 2009) (Both)
To raise the capability of classifiers in image distortions, 2017 (Tschentscher et al. 2017)	Enhance the efficiency of classifier in image distortion such as rain, cloud and snow	Enhance the efficiency of classifier and reached the classifier efficiency to 99.72%	Fog, sunny, rain, snow (environmental impact)	Unreal Engine 4 (AI)	Real and virtual self generated images (Both)
Plant recognition system by DL technique, 2019 (Kazerouni et al. 2019)	Make plant recognition system using DNN in environmental challenging conditions	The accuracy of their technique is 99.5% as compared to other state-of-the-art DL techniques (Bay et al. 2006)	Wind and Rain (environmental impact)	DL (Convolutional Neural Networks)	Modern Natural Plant Dataset (MNPD) (Non-synthetic)
A framework for improving the performance among lane and 2D object detection under bad weather conditions in terms of both low memory and accuracy, 2021 (Lee et al. 2021)	A strategy is presented that minimizes image enhancement’s detrimental effects while improving high-level task performance to a task-driven end	A task-driven enhancement network is developed for less memory and computational cost, making it suitable for embedded systems of autonomous cars	Rain and haze (environmental impact)	Dense Convolutional Network (Huang et al. 2017)	TuSimple dataset, KITTI (Geiger et al. 2012), and RID dataset (Li et al. 2019) (Both)
A gradient flow based deep residual network, for improving scene visibility degraded by fog, 2021 (Suganya and Kanagavalli 2021)	Transmission map determination and foggy haze removal using residual network based on the estimation of the ratio between transmission map and foggy image	Associating the input of haze and defog images having maximum accuracy by generating the transmission map	Fog (environmental impact)	Deep residual network	Image Defogging Research at LIVE [80], FRIDA3 (Tarel 2022) (Both)
A transfer learning based general image enhancement model used for images degraded by specific natural environmental parameters such as rain and snow, 2021 (Yin and Ma 2021)	Comparing the efficacy of general image enhancement model for rain, snow and underwater with the individual image enhancement model for rain, snow and underwater	Address the general issues of limited training dataset and computational complexity	Rain and snow (environmental impact)	Transfer learning	Outdoor-Rain (Li et al. 2019), Snow 100K (Liu et al. 2018), Underwater Image Enhancement Benchmark (UIEB) (Li et al. 2019) (Synthetic)
An end-to-end model based on transformers that can restore an image damaged by any weather condition, 2022 (Valanarasu et al. 2022)	Developing a single encoder-decoder based transformer that provides an efficient solution to the problem of adverse weather removal	To reduce the number of transformer parameters that used multiple encoders specific to each weather removal task	Rain, fog, and snow (environmental impact)	Trans-Weather (Transformer)	Test1 dataset (Li et al. 2019, 2020), the RainDrop test dataset (Qian et al. 2018) and the Snow100k-L test set (Both) (Liu et al. 2018)
Comparison of three variants of You Only Look Once (YOLO) object detection algorithm on vehicle detection models, 2022 (Boppana et al. 2022)	The purpose of this study is to investigate single-shot detection, which is YOLO algorithm with its variations to detect vehicles in real-time	How to find bench marking results for improving vehicle detection algorithms	Rain, snow and haze (environmental impact)	YOLO	AAU Extreme Weather Dataset (Non-synthetic) (Bahnsen and Moeslund 2018)

**Fig. 6:**
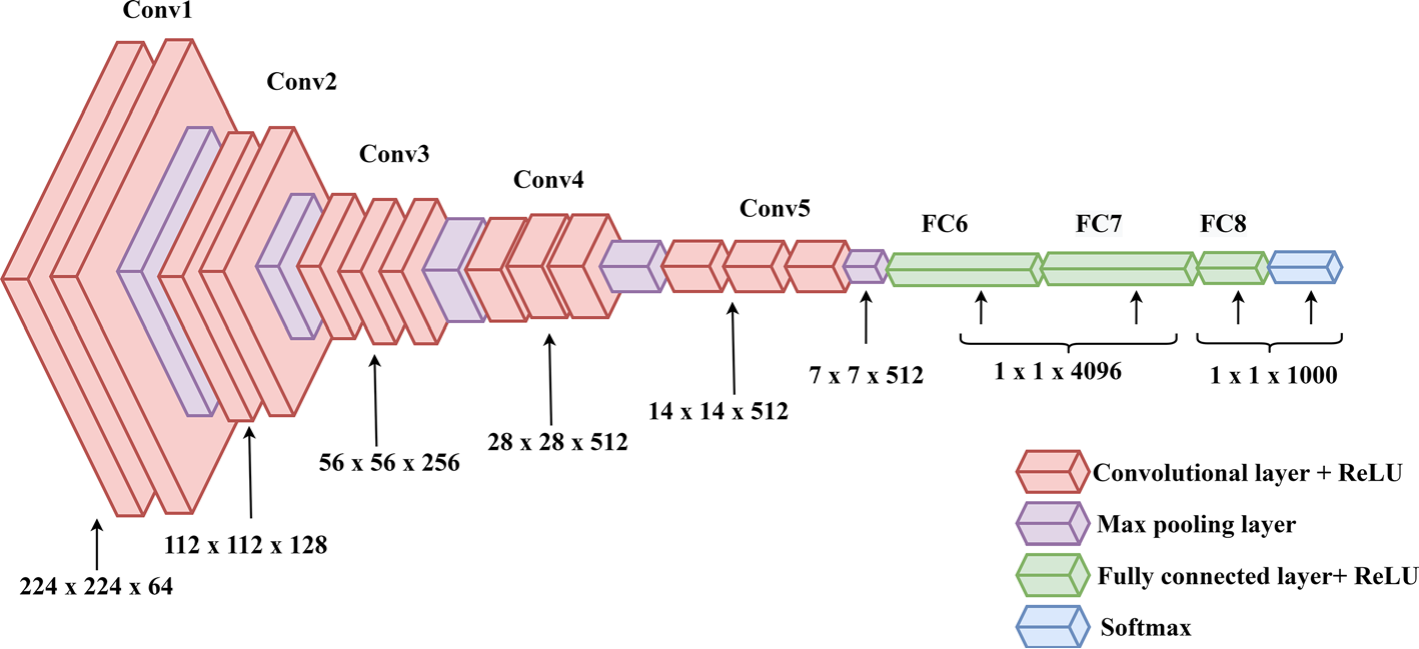
Basic structure of VGG16 CNN Model

There are diverse DL-based approaches such as Convolutional Neural Network (CNN) (Albawi et al. 2017), Recurrent Neural Network (RNN) (Pascanu et al. 2013), and many more which are utilised to analyse and reduce the impact of environmental and camera distortion parameters. To illustrate, the basic structure of VGG-16 CNN is delineated in Fig. 6. CNNs are a variety of artificial neural networks used in image and text recognition (Jain et al. 2007), classification, segmentation, face detection (Matsugu et al. 2003) and others. A typical CNN consists of three layers: (i) a convolutional layer, (ii) a pooling layer, and (iii) a Fully Connected (FC) layer. Feature extraction is performed by the first two layers which are convolution and pooling layers. FC layer do mapping of extracted features to final output. The convolutional layer plays a crucial role which performs mathematical operations like convolution. In Fig. 6, convolution layer 1 is fed with 224 x 224 RGB image input. black block represents the max pooling layer which calculates the maximum value in every feature map patch. Following five convolutional layers, there are three FC layers. Lastly, there is a soft-max layer that performs multi-class classification (Simonyan and Zisserman 2014).

The CNN mentioned in Fig. 6 is one example of a DL-based technique that is used for the analysis and reduction of environmental and camera impacts. There exist various other DL techniques which are discussed in the following sections.

### Assessing environmental impacts on IoT images

For improving the reliability and effectiveness of IoT applications which are mentioned in Sect. 3, it is pivotal to assess the environmental impacts on IoT images. As alluded before, there are various weather conditions such as rain, shadow, darkness, etc. that can affect the quality of IoT images. The efficacy of DL algorithms is affected by such challenging conditions because the impacted images contain many distortions that increase the uncertainty of DL algorithms.

To analyse the impact of rain, sun, and fog, Tschentscher et al. (2017) implemented a new simulation system using Unreal Engine 4 to enhance the efficiency of the traditional parking system. The traditional parking system was based on an embedded system without any use of smart cameras. There was an insufficient amount of data on the traditional parking system. Therefore, the authors created a simulation model that can be used to capture data considering different environmental and camera impacts. Because a large amount of data is required for the training of DL models. For this, a portrait of a parking system is made that contains many rows. The authors fostered a simulated smart camera that executes all highlights of a genuine camera such as lens blur and lens distortion.

The camera is used to observe and store three-dimensional information about the scene. Most of the features of the camera are similar to the real one such as it creates lens distortion and various aperture sizes. Recorded images (grey or coloured) are then stored in memory. Then the image processing system is implemented, which can fetch the images from the memory. The particular used Unreal Engine 4 is a game engine that is used to compare a real scenario with its respective simulated one. To provide a real touch to the system, the coating of materials is done by Physically-Based Rendering (PBR). Because PBR-based scenarios are more realistic compared with their corresponding non-PBR-based counterparts. There is a trigger volume to hear whenever any car is entering or leaving the parking space. Based on that the parking details are written to the log file such as parking space ID and information about the parking space whether it is empty or occupied.

The main advantage of reproduced conditions is the full command over ecological boundaries. The authors covered every situation such as a difference in climate and diverse daytime lighting conditions, etc. This system has control of exposure, focal length, aperture opening, and focus distance while capturing images. Some post-processing steps are implemented to reduce the effects of lens distortion, motion blur, and image noise. The lighting conditions are adjusted in three different weather conditions such as sunny, foggy, and cloudy. The accuracy of the video-based parking guidance system is less in cloudy and sunny environments. For the foggy environment, it depends on the density of the fog increases with an increase in the camera distance.

Snow creates one of the difficult challenges while capturing images in the outdoor environment. Therefore, the accuracy of their system in the snow is 70%. Overall, the algorithm can work in difficult weather and lighting conditions, where a classifier is trained to turn on or off lights accordingly. The performance of the new parking classifier modelled with the simulated data is verified with various recorded real scenarios. The results show the classifier is capable of handling challenging conditions such as snow and rain but the accuracy is low (70% in snow and 93% in rain).

As mentioned before, while dealing with snow, the accuracy of this system is 70%. Also, the video captured from the simulated environment was compared with its respective real-world scenarios. However, they have not leveraged this simulated video to demonstrate the improvement of the efficacy of the real-world/traditional parking guidance system. Hence, to improve this system, there is a need to train the model using this simulated video data for the parking guidance system and test the trained model using real/live parking traffic conditions data under different traffic scenarios and environmental impacts.

Hence, to make the parking system more flexible and cover more weather conditions such as fog, a reliable system based on the DL algorithm executed in edge devices was proposed for smart parking surveillance systems. The impact of weather conditions, temperature, and different timings of the day are considered. The metrics that are used to indicate the goals for performance of their system are: smartness is the automatic detection and pattern recognition in a parking garage scene; efficiency is the online processing, and reliability is the detection performance that is reliable in all kinds of environments. For testing the proposed system, a busy Angle Lake parking garage having 1160 available parking slots is used. IoT devices are installed on the sixth floor in an outdoor environment to analyse the performance of the system in dynamic outdoor environment conditions. The authors considered mainly four environmental parameters such as cloud, rain, fog, and sun. First, the system is developed and tested in a lab environment, and then it is put into use in a real-world parking garage for three months. A computer vision-based algorithm is used to detect objects and the modified SORT algorithm (Bewley et al. 2016) is utilised on the server side to decrease the load on the edge side. The system outperforms in the cloudy conditions compared with the other three conditions (rain, fog, and sun) with an accuracy of 97.5% on weekdays and 99.2% on weekends. It performs well in the rainy environment in relation to the sunny and foggy environments having 93.75% accuracy on weekdays and 96.2% accuracy on weekends. The accuracy of the system is maximum at night as compared to daytime and other weather conditions mentioned above because the total number of occupied parking spaces and traffic volume were both lower at night. The accuracy of the system is less in foggy and sunny weather conditions because, in sunny weather, there are huge shadows and reflections toward the camera lens. The results show the least accuracy in a foggy environment. Because fog creates more impact on image quality compared with the other three factors (Ke et al. 2020).

Therefore, to improve the accuracy of the system in a foggy environment, Khan et al. (2019) introduced a deep CNN-based approach for detecting smoke in a normal and foggy IoT environment. Detecting smoke in smart cities is not an easy job especially when smoke is in its initial stages or in uncertain environments such as fog. The systems which use traditional feature extraction approaches produce a large number of false alarm rates and less accuracy in smoke detection. Also, existing methods are expensive and not suitable for real-time. The authors found out that VGG-16 is better and they fine-tuned the last layer of architecture from 1000 to 4 classes to perform planned classification of smoke and non-smoke in foggy and normal environments. The authors created their benchmark smoke dataset and compared the performance of their DL-based model with that of existing DL-based techniques such as Googlenet and Alexnet. Their proposed approach achieves much better accuracy (97.72%) compared with existing state-of-the-art methods. However, the authors just considered fog as an uncertain environmental parameter but detection accuracy in other environmental parameters such as shadow, sun, and others is not discussed. The solution for these issues is to gather data under different environmental conditions and test their system with collected data and exploit the insights received from the results to improve the given approach.

Similar to smart transportation, the need to analyse the quality of IoT images for smart agriculture is also essential. Because in smart agriculture, leaves are an important part of plants. There are a lot of factors that can change the shape and size of leaves. It can be mainly due to environmental parameters such as a change in weather conditions (e.g., snow, sunlight, rain) which results in the deformation of leaves or dried leaves. Also, windy weather affects the clarity of images. Most of the images would be blurry which can be due to lens blurriness and results in the deformation of leaves. Another factor is fog due to which the contrast of an image is changed. And there will be light scattering and blocking due to some water droplets. Rain also leads to an increase in the intensity of images.

Besides weather, lighting conditions can also hugely impact the imaging process. This can be attributed to the fact that the images taken in the different parts of the day such as in the morning or the afternoon have a lot of variations in brightness and intensity. All these above factors can change the image content to a great extent and make images much more complex.

All the challenging conditions discussed such as weather types (rain, wind, temperature, humidity), complex backgrounds, time of photographing and change of light intensity make plant recognition difficult.

For this reason, Kazerouni et al. (2019) presented an automatic plant recognition technique. In this technique, DL is used to identify plants in the above-mentioned challenging conditions. The authors used CNN in which the first five layers are convolutional layers and the other three are FC layers. They used their model on the Modern Natural Plant Dataset (MNPD) in which the training dataset contains 800 images and the test dataset consists of 200 images in comparison with previously existing methods (Kazerouni et al. 2017, 2017) in which 664 and 336 images were used as training and test set images respectively. The accuracy of previously existing systems presented in Kazerouni et al. (2017) and Kazerouni et al. (2017) is 94.9 and 93.9, respectively. The overall plant recognition accuracy of this technique under challenging conditions such as rain, wind, temperature, humidity, and light intensity is reported as 99.5%. This system is a distance-independent system which does not depends on the distance between the camera and the plant. Because of the deep CNN structure, this technique is regarded as the most effective.

However, only four species of plants are considered for the recognition process, and only two environmental parameters such as rain and wind, and camera parameters such as distance are considered in the results. There is a need to consider more species of plants such as rice and cassava that are difficult to recognize (Hassan and Maji 2022) and other environmental parameters such as shadow, darkness, and fog.

Image noise is also one of the environmental distortions that can happen while capturing IoT images (Buades et al. 2005). An increase in temperature leads to image noise. To analyse how image quality affects computer vision applications, (Dodge and Karam 2016) evaluated the impact of noise by DNN models. For this experiment, they used four different neural networks. The first network is Caffe Reference Model (Jia et al. 2014) which is an execution of AlexNet network (Krizhevsky et al. 2012). The second model is the VGG-CNN-S model (Chatfield et al. 2014), similar to Caffe Reference Model, but it has greater efficiency compared with Caffe Reference Model. The third model is the VGG-16 model (Simonyan and Zisserman 2014) which has more convolutional layers than the previous two models. The last model is GoogleNet model (Szegedy et al. 2015) which uses fewer parameters than others. The performance of VGG-16 and GoogleNet is better compared with the Caffe and VGG-CNN-S networks in noise. This is because of their deeper structure. Deeper structure involves multiple layers in the model. Multiple layers have the advantage of learning features at different abstraction levels. A deeper structure allows the networks more room to learn features that are not affected by noise. Despite JPEG and JPEG2000 compression distortions, the networks are surprisingly resilient. Performance drops only at very low-quality levels (quality parameter less than 10 for JPEG and PSNR less than 30 for JPEG2000). Note that the quality values are in the range 0–100 for JPEG and JPEG 2000, where 0 indicates the lowest quality value and 100 indicates the highest quality value. Given that the compression level is sufficient, deep networks can be reasonably confident of performing well on compressed data. The disadvantages of deeper structures are more power consumption and a time-consuming training process. It takes a lot of time which consequently leads to an increase in run time and the requirement for good memory GPU. Moreover, if the network is very deep, this may result in vanishing or exploding gradients (Tian et al. 2020). Overall, they concluded that the VGG-16 network is more robust against noise than other models. The synthetic Gaussian noise is added to the colour component of each pixel to analyse the impact. However, as per (Dodge and Karam 2016), synthetic Gaussian noise cannot capture all types of noises from the deployment environment which is the shortcoming of this method. Hence, for the accurate validation and performance analysis of those DL-based models, there is a necessity to use the datasets captured under many different real-world noisy environmental scenarios.

For assessing the environmental noise more accurately, since the Gaussian noise is generally adopted to represent the environmental noise, the impact of Gaussian noise is assessed by using DNN classifier (Zhou et al. 2017). Gaussian noise is generally adopted to represent the environmental noise, and the impact of Gaussian noise is assessed by using the DNN classifier. However, the other feasible alternatives could be unsupervised Deep learning models such as Deep Belief Networks. The good results are reported on a noisy dataset called n-MNIST, which contains Gaussian noise. We believe that the noisy versions of MNIST and Bangla datasets will help researchers better apply and extend the research on understanding representations for noisy object recognition datasets. The advantage of using this particular approach is improvement in the accuracy when classifying distorted images. This improvement indicates that classification results on distorted images can be close to the results on original images. Authors used more datasets such as different types of small datasets (LeCun et al. 1998; Krizhevsky et al. 2009) and a large datasets, namely Imagenet dataset (Deng et al. 2009). While studying the impact of noise, they noticed that the performance of the DNN classifier degrades. For this purpose, they re-trained their network including images containing noise in which a large training dataset is required. They also changed some first layers with the degraded images and made adjustments in the low-level filters of DNN to match the features of degraded images. Therefore, to improve the performance of their model, they re-trained and fine-tuned datasets. For a high level of noise, better results are given by re-training for distorted datasets. For low levels of noise, good results are provided by fine-tuning and re-training together. Fine-tuning is preferable because it has the advantage of saving computational time. Because fine-tuning takes less training time in comparison with re-training the model. (Zhou et al. 2017).

The effect of Gaussian noise on DNN was also analysed by Basu et al. (2017). The additive white gaussian noise was generated by adding noise with a Signal-to-noise-ratio (SNR) of 9.5. 9.5 is a standard SNR. The advantage of using SNR=9.5 is that it simulates background clutter well. The noise which is represented by this SNR value is similar to noise that is caused by electrical variations in cameras, television static, etc in real-world applications. However, the disadvantage is that this system can only perform well under the impact of noise at SNR greater than or equal to 9.5. The alternative way to add Gaussian noise is with a Gaussian distribution function with varying values of mean and Standard Deviation (SD). The authors achieved an improvement of 69% with their learning framework based on probabilistic quadtrees compared with previous traditional deep belief networks on MNIST, n-MNIST, and the noisy Bangla datasets. Therefore, in these two above-mentioned noise analysis methods, authors synthetically generated noise but not from the natural source of the noise. Hence, firstly, there is a requirement for the database in the real environment to analyse the impact of different environmental parameters. Secondly, to address the issue of noisy datasets, the authors’ future research direction is to plan to examine various pooling techniques like SPM (Lazebnik et al. 2006) and certain sparse representations such as sparse coding (Lee et al. 2006). So far, we present the environmental impact assessing techniques on IoT images. However, research shows (see Table 2) camera lens distortions affect IoT images considerably. The following section details the techniques for analysing the camera lens distortion impacts on IoT images.

### Assessing camera lens distortion impacts on IoT images

There are different types of camera impacts such as lens blur, lens dirtiness, and barrel distortion that happen while capturing IoT images. Nevertheless, the current literature exhibits DL techniques have been applied to analyse the impacts of lens blur (Zhou et al. 2017; Dodge and Karam 2016) and lens dirtiness on IoT images as shown in Table 2. For example, lens blur can happen due to the movement of the camera or when the camera is not in focus mode (Nejati et al. 2016; Pomponiu et al. 2016; Do et al. 2014). Camera lens blur leads to the loss of high-frequency components and thus results in low resolution.

To analyse the impact of lens blurriness and JPEG compression, Dodge and Karam (Dodge and Karam 2016) used Caffe Reference Model (Jia et al. 2014), VGG-CNN-S model (Chatfield et al. 2014), VGG-16 model (Simonyan and Zisserman 2014) and GoogleNet model (Szegedy et al. 2015) for their experiments. To create the blur effects, the Gaussian kernel is used and the SD of the Gaussian is varied from 1 to 9. Filter window size is set to four times the SD. Even though authors used fixed window size, however, the window size determines the extent of filtering. Larger sizes generally result in greater filtering. Larger windows require more processing and produce higher levels of blurring. For example, if the filter window size is set to eight times the SD, it would lead to high levels of Gaussian blurring than when the window size is equal to four times the SD. Blur creates a slight change in the first convolutional layer. Contrary, the response to the last convolutional layer differs significantly when blur is introduced in the first layer. The result of this is that small changes in the first layer are propagated to create larger changes at the higher layers. It shows that the reduction in the performance of DNN under blur is not limited to a particular model, nevertheless, it is common to all considered DNN architectures. Their analysis shows all the models are very sensitive to lens blurriness. However, VGG-16 network appears more robust than the other networks. In this method, authors considered only the lens blur effect on the performance of DNN but other camera impacts such as lens dirtiness and barrel distortion are not considered.

The impact of blurriness is also assessed by DNN classifier (Zhou et al. 2017). The applications utilised in this research project have different types of distortion such as motion blur, defocus blur and a combination of them. Authors used various datasets (LeCun et al. 1998; Krizhevsky et al. 2009) such as different types of small datasets (LeCun et al. 1998; Krizhevsky et al. 2009; Deng et al. 2009) to study the impact of lens blurriness. They analysed that the performance of the DNN classifier degrades with an increase in the impact of lens blur. Retraining and fine-tuning are done to reduce the Top-1 error rate. Experimental results a reduction in the Top- 1 classification error rate of distorted images from 53.1% to 47.7%.

In Basu et al. (2017), the effect of lens blur is analysed in which the authors achieved an improvement of 26% for lens blurring for the n-MNIST dataset compared with previous traditional deep belief networks. The main advantage of this method is that it also considers a challenging condition such as reduced contrast in which the efficiency is improved by 16%. Besides these challenging conditions, there are other challenging conditions such as camera distance and movement in the camera, which result in the degradation of the quality of images. DL algorithms are greatly influenced by these camera perturbations. Even small camera movement results in the misclassification of trained neural networks. Even small camera movement in any directions including either x-axis or y-axis. These challenges can create more serious problems when used in safety-critical systems such as autonomous vehicles. For example, sometimes a stop sign can be regarded as a minimum speed limit sign which can cause serious accidents. YOLO detector is used for the measurement of the detection rate of different traffic signs contained in IoT images. Photos with varying distances are taken and analysed so that the rate of distortion increases with an increase in the distance. Results show that the change in the angle can also impact the effectiveness of the models (Lu et al. 2017). However, the proposed DL algorithm has been tested with stop signs only. Moreover, traffic signs are represented in different sizes and different colours. Besides lens blurriness impact, the camera distance affects the quality of its images differing from traffic sign to traffic sign. Therefore, to show the effectiveness of this technique for autonomous vehicles, it needs to further investigate the performance using various traffic signs under the different camera and environmental impacts. For example, different camera impacts include lens dirtiness and barrel distortion etc. Different types of environmental impacts are also mentioned in Sect. 1.

Temel et al. (2018) analysed the impact of lens dirtiness and lens blur. The authors introduced the Challenging Unreal and Real Environments for Object Recognition (CURE-OR) dataset, which consists of 1,000,000 images of 100 objects of various sizes, colours, and texts. Under challenging conditions such as lens blur and lens dirtiness, it is demonstrated that Amazon Rekognition and Microsoft Azure Computer Vision performance significantly degrades. The gaussian operator is used to obtain blur images. Dirty lens patterns have been overlayed on original images to create dirty lens images. Top-5 accuracy metric and confusion metric was employed to show the recognition performance. Top-5 accuracy means that any of the top five most probable answers match the expected answer. The challenging conditions such as lens blur and dirty lenses lead to Amazon’s and Microsoft’s accuracy dropping below 1%. Because a dirty lens pattern with different weights corresponds to a challenge level, effectively blocks the edge and texture in an image. To conclude, based on the analysis of the categorical misclassifications, recognition performance degrades as the challenge level increases for each challenge category (lens dirtiness and lens blur). In this lens blur analysis, the recognition performance was measured by only top-5 accuracy and do not mention top-1 accuracy. And the Gaussian operator is used to obtain blurred images, but the authors do not examine the impact on naturally blurred images which would restrict its application in real-world environments. Also, other camera impacts such as barrel distortion have not been covered. Therefore, there is a requirement to capture databases in more realistic environments.

So far, we provided the solution for RQ-2 where the DL techniques focusing analysis of environmental and camera impacts on IoT image capturing are presented. However, there exist some DL approaches for reducing both environmental and camera impacts on IoT images that correspond to the solution of RQ-3. These approaches are presented in the following sections.

## Reducing impact on IoT images using DL

Table 2 and the above discussion depict that dynamic outdoor environmental parameters such as fog, rain, snow, and camera parameters have adverse impacts on image processing applications (e.g., transportation, agriculture, city, and health). Hence, there is a need to minimise the impact of such parameters on IoT images because these parameters limit the potentiality of application systems.

### Reducing environmental impact on IoT images

From the early onset of the research on reducing the environmental impact on images, there exist many techniques that are mainly not DL-based (Narasimhan and Nayar 2002; Shwartz and Schechner 2006; Fattal 2008; Tan 2008; Tarel and Hautiere 2009; He et al. 2010). For example, haze is mostly created by weather conditions. It is one of the environmental parameters due to the attenuation in the light, which results in the reduction of image quality. The decrease in image quality leads to loss of information and improper recognition of objects. Various studies (Narasimhan and Nayar 2002; Shwartz and Schechner 2006; Fattal 2008; Tan 2008; Tarel and Hautiere 2009; He et al. 2010) have done to reduce the impacts of haze by different methods of physical equations like atmosphere scattering model (Gu et al. 2017) and improvement in contrast techniques. Although these methods achieve dehazing, they take a lot of computational time and are inefficient to remove haze from every part of the image.

Up to now, we present non-DL-based environmental impact reduction techniques. However, with the advancement of neural networks, especially the DL algorithms, a few relevant DL-based techniques are available in the current literature for reducing environmental impacts. The pioneering works in this area are the approaches developed using DL algorithms (CNN), neural network filters, and dense correspondence-based transfer learning. These algorithms decrease the impact of rain, shadow, darkness, clouds, snow and haze on IoT images and the images captured by traditional cameras.

A combined approach of DL (neural network filter) and fuzzy inference system to tackle the problem of hazing in images is presented in Wang et al. (2016). Haze primarily occurs when light is attenuated. It is a nonlinear problem to estimate the attenuation of light. For this reason, a fuzzy inference system is designed to estimate the variation in the intensity of light. Hence, the authors used this combined approach to effectively remove the haze effect. Because the fuzzy inference system is based on the human brain’s ability to express word ambiguity. The neural network emulates the brain’s organizational structure and is capable of learning. In this method, factor *e* is used as a performance metric. It is the capability to restore the ratio of edges that are not visible in the original image. It is 0.06 in comparison with other techniques whose value for e is $-$0.06 (Tan 2008), $-$0.01 (Tarel and Hautiere 2009) and 0.01 (He et al. 2010). Along with haze which can impact the quality of images, factors such as shadow, darkness, rain, snow and cloud can also impact the quality of images. As alluded to before, in the field of transportation systems, there is a problem to understand traffic scene images on rainy, cloudy, dark and sunny days.

To reduce the impact on traffic scene images, series of experiments were done using a challenging dataset consisting of a total of 1828 images having different weather conditions. A Dense Correspondence-based Transfer Learning (DCTL) technique was used by Shuai et al. Di et al. (2018) which consists of three main steps that use:

1. a fine-tuned convolutional neural network to extract deep representations of traffic scene images;
2. cross-domain metric learning and subspace alignment to construct compact and effective representations for cross-domain retrieval; and
3. cross-domain dense correspondences, and a probabilistic Markov random field to transfer annotations from the best-matching image to the test image.

Traffic scene data sets are quite different from the data sets used to pre-train CNN in the authors’ case. Because of this, authors fine-tune the pre-trained CNN using traffic scene data. Depending on the weather and illumination conditions, traffic scene images may have dramatically different appearances, and the extracted deep features may also not be consistent. Domain adaptation allows for overcoming this challenge. For instance, the terms “sunny day", “cloudy day", and “snowy day" belongs to domain A; while “night", “rainy night", and “foggy day" are in domain B. Annotation information on scene images is transferred from the cross-domain best-matching image to the test image based on the dense correspondences established between them. Different images in different domains usually have different appearances due to differences in weather or illumination conditions, but they share a similar spatial layout. Thus, if proper correspondences are created, the annotation information on scene images in domain A can be transferred to scene images in domain B. The dense correspondences are constructed using Scale-Invariant Feature Transform (SIFT) flow (Liu et al. 2010, 2011) and the annotation information is transferred using a Markov random field model. For all these challenging scenarios, the DCTL technique performs better compared with other state-of-the-art approaches such as Geodesic Flow Kernel (GFK) (Gong et al. 2012) and Subspace Alignment (SA) (Fernando et al. 2013). It is because of the fine-tuning of CNN to extract deep features and subspace alignment for constructing compact representations to retrieve the cross-domain best-matching image. The 12.57% improvement is seen in the detection accuracy of traffic signs with the fine-tuning of CNN’s.

However, the DCTL technique is not adequate as it is insufficient to compensate for possible variations in illumination. Consequently, an advanced method (Di et al. 2021) is proposed recently to address the problem of images captured in rainy and night scenes without pixel-level human annotations. For these experiments, the authors created a new dataset comprising different 7000 images. The proposed method outperforms the previously mentioned algorithms (e.g.,“A dense correspondence-based transfer learning approach” (Di et al. 2017) and “Dark Model Adaptation” (Dai and Van Gool 2018)) which have the problem of domain adaptation for semantic segmentation of rainy night images. This issue is addressed in this technique (Di et al. 2021) using adversarial learning, which does not require pixel-level human annotations.

There are varied DL methods such as removal of rain (Ren et al. 2019; Ahn et al. 2022), removal of fog (Wang et al. 2019), removal of haze (Wang et al. 2022; Feng et al. 2022) and many more. However, these methods cannot work effectively to manage comprehensive environmental conditions and they just worked on the synthetic database rather than real images.

To solve this issue of artificially generated environmental parameters on images, Wei et al. (2019) proposed a semi-supervised transfer learning for the removal of rain from the images. Authors used 64$\times$64 synthesised rainy/clean image patch pairs as supervised training data, which are the same as those used by the baseline CNN method. To formulate the loss function on supervised samples, the authors followed the network structure and negative residual mapping skill of DerainNet (Fu et al. 2017). Real-world rainy images were used from dataset (Wei et al. 2017) and Google image search. The unsupervised samples were obtained by randomly cropping one million 64$\times$64 image patches from these images. Unlike deep learning methods that only use supervised image pairs with/without synthetic rain, authors put real rainy images, without the need for their clean ones, into the network training process. They also used real rainy images for the network training process. Both supervised and unsupervised images of rain are used to train a CNN. The difficulty of collecting training samples and overfitting training samples are particularly alleviated by their method in comparison to other DL methods designed for this task. Nevertheless, this model is not capable of handling all rainy images which are extremely complex (images containing high or extremely high-level rain impact). As the authors did not experiment using a dataset containing heavy rain, a future project, is aimed to show the efficacy of their method to reduce the impact of heavy rain (Wei et al. 2019). Also, only environmental parameter such as rain is covered in this experiment indicating other environmental parameters such as shadow, darkness, wind, and fog also need to be considered.

To address this issue in which authors just focused on the removal of rain, Chen et al. (2020) devised an adaptive noise removal tool based on the VGG. The proposed adaptive noise removal tool improves the quality of images with the optimised algorithms. The method is beneficial for the removal of sunny, foggy, cloudy, and rainy images. However, snowy weather is still in their future works which need to be considered. Until now, environmental impact-reducing techniques on IoT images are presented. The techniques to reduce the camera lens distortion impact for IoT images are explained in the forthcoming section.

### Reducing the impact of camera lens distortions on IoT images

As mentioned in Section 3.2, there are various types of camera lens distortions such as lens blur, lens dirtiness, barrel distortion, or fisheye distortion.

There exist different works for lens deblurring such as Park et al. (2015) introduced a method called the “self example image enhancement” method to alleviate the effect of image blurriness occurred by the camera lens.

Recently, Bhonsle (2021) states that the Lucy-Richardson algorithm is utilised for the deblurring of images, although, this method can be used to deblur the images up to a certain extent.

To overcome this complication, a Krill Herd method was employed and a comparison of this method with pre-existing on the basis of PSNR, SNR, and SSIM proved the efficacy of the system (Bhonsle 2021).

In Park et al. (2015), barrel distortion correction is done by an orthographic projection model based on the parabolic equation. An algorithm for real-time applications to correct barrel distortion using MATLAB and BF561 DSP is presented in Awade et al. (2016). The main shortcoming of this method is that the distortion correction factor is adjusted manually according to image distortion. However, for a faster and more automatic process, the correction factor could also be adjusted automatically according to the focal length of the lens. Note, the camera lens distortion reduction approaches presented up to this point are non-DL-based. However, to our knowledge, there exist few DL-based methods for camera lens distortion reduction that are presented in this section.

As alluded before, to address the issue of manual adjustment of the distortion correction factor, Liu et al. (2018) developed a DL-based model entitled “smart unstaffed retail shop schemes.” The model has a front-end layer that consists of a camera and an intelligent unmanned retail container. The authors used a dataset with the composition of 11,000 images having different kinds of scenarios. A DL-based method, namely Mask-Regional Convolutional Neural Network (MASK-RCNN) is utilised to train their model. The model achieves 96.7 % recognition accuracy for the test set of images. To improve the efficacy of the method introduced by Liu et al. (2018) for fisheye or barrel distortion reduction, there exist some other methods (Li et al. 2011; Park et al. 2009; Wang et al. 2015). These methods use the spherical projection (Bourke 2010) technique for barrel distortion reduction which makes the system less computationally complex.

Das et al. (2017) also showed that DNN plays a significant role in solving the problems of machine learning, particularly in image recognition processes. Latest studies indicate that DNN are at high risk of adversarial generated instances that can confuse DNN but it seems fine for human beings. To compensate for these effects, JPEG compression is an effective way of pre-processing to remove the noise. Because it can remove high-frequency components. However, noise could be due to various impacts such as weather-changing parameters and camera lens distortions. The definition of noise is not clear in this approach.

Until now, we have presented the solution for RQ-3 where the DL techniques have focused on reducing the environmental and camera impacts on IoT image capturing. Even so, there are still a few research challenges that need to be addressed, which are discussed in the next section.

**Table 3:** Future research challenges associated with DL techniques for the analysis and reduction of environmental and camera impacts and suggested ways to address those challenges. Here, Env, Cam, Anal, and Red represent environmental, camera, analysis, and reduction, respectively

Approach/Year	Future research challenge	Ways to address challenge	Impact type		Impact process	
			Env	Cam	Anal	Red
Implementation of a simulation system using Unreal Engine 4 to analyse the impact of rain, sun, and fog (2017) (Tschentscher et al. 2017)	There is no attempt made to use this simulated video to demonstrate the effectiveness of the real-world or traditional parking guidance system(s)	The simulated video data needs to be used for training the parking guidance model. The performance of the model is required to be tested under various environmental impacts based on real parking traffic scenarios	$\checkmark$	$\times$	$\checkmark$	$\times$
To analyse the effect of lens blur on DNN (2017) (Lu et al. 2017)	the experiments are limited to recognising stopping signs in traffic databases. This limitation is a concerning situation in many real-world circumstances, especially for autonomous vehicles	With the usage of all different road and traffic signs used in traffic control systems, the efficacy of the proposed method needs to be tested before applying it in intelligent transportation systems involving autonomous vehicles	$\times$	$\checkmark$	$\checkmark$	$\times$
Deep CNN-based approach for detecting smoke in a normal and foggy IoT environment (2019) (Khan et al. 2019)	Fog is regarded as the only uncertain environmental parameter. However, other environmental impacts such as shadows and sunlight are not addressed	The system needs to be tested under different environmental conditions. The insights gained from these tests can help improve the system	$\checkmark$	$\times$	$\checkmark$	$\times$
Automatic plant recognition technique using DL (2019) (Kazerouni et al. 2019)	Not many (only four) plant species and environmental parameters (two) such as rain and wind are considered in the plant recognition process	Plant species such as cassava that are difficult to identify (Hassan and Maji 2022) and other environmental parameters such as shadow, darkness, and fog need to be included	$\checkmark$	$\checkmark$	$\checkmark$	$\times$
Semi-supervised transfer learning for the removal of rain from the images (2019) (Wei et al. 2019)	It would be difficult to handle complex rainy images with this model and only rain is considered as environmental parameter	The model can be trained using difficult rainy conditions and other environmental situations created by other different environmental parameters	$\checkmark$	$\times$	$\times$	$\checkmark$
Analysed and compared three variants of the YOLO object detection algorithm based on vehicle detection models, 2022 (Boppana et al. 2022)	How to measure the amount of degradation occurs in vehicle detection model and how much it suffers from deterioration of input images	To develop a mathematical function that can measure the correlation between the quality of images and the performance of the vehicle detection model	$\checkmark$	$\times$	$\checkmark$	$\times$
An end-to-end model based on transformers that can restore an image damaged by any weather condition, 2022 (Valanarasu et al. 2022)	Only environmental parameters such as rain, snow and fog are considered	Other environmental impacts such as shadow, darkness can be included in the experiments	$\times$	$\checkmark$	$\checkmark$	$\times$

## Further research challenges

Even though there exist many techniques available in the literature, impact analysis and reduction techniques including the DL-based approaches for IoT images are still at an embryonic stage.

Table 3 presents future research challenges concerning particular techniques for the analysis and the reduction of environmental and camera parameters including their impact types. For example, a further research challenge for the automatic plant recognition techniques is that the authors examined the effects of rain and wind on only four plant species. The recommended ways to address future research challenges are also mentioned in this table. The recommended way for addressing the above-mentioned research challenge is to work on plant species like cassava that are difficult to identify (Hassan and Maji 2022).

Moreover, there are general ways to advance research in this area. Some of the possible future research issues and their recommended solutions are presented as follows:

1. Objective quality assessment techniques (e.g., PSNR, SSIM, ORB, MSE, SC) (Kaur et al. 2022) and some DL-based quality assessment techniques (Dodge and Karam 2016) are not consistent. Because these techniques cannot capture impact levels accurately and represent the impact level consistent with human perception for all environmental and camera impacts. These inconsistent impact levels demand the development of a new image quality assessment technique. The new technique could be either based on DL or a statistical approach and should consider the characteristic aspects of both environmental and camera impacts on IoT images more accurately.
2. The recent trends for the industry, business, and governmental organisations are to use IoT technologies to capture the value from data. These trends include big data usage, including video and unstructured data, and introducing innovative products and cost-effective digital or automatic services for better customer/user satisfaction and economic boost. However, benchmark IoT datasets on different environmental and camera impacts are not available with current research communities. The unavailability of such datasets is one of the barriers to developing effective image quality assessment techniques and assessing both environmental and camera impacts on IoT images accurately.
3. The performance analysis of existing DL-based distortion reduction techniques for all possible environmental and camera distortion impacts on the IoT images needs to be advanced further. For example, (Tschentscher et al. 2017) covers only the impacts of fog, sun, rain, and snow but not other environmental impact factors such as wind, shadow, and darkness. Likewise, the approach presented in Kazerouni et al. (2019) emphasises only wind and rain. However, the rest of the environmental parameters such as shadow, snow, and fog while capturing IoT images are not considered. Similarly, even though Liu et al. (2018) have reduced the impact of barrel distortion, other camera parameter impacts such as lens blurriness and dirtiness are not considered. The information presented in this further research indicates that the existing distortion reduction techniques are not taking all the environmental and camera lens distortion impacts into consideration.
4. All DL techniques are not effective enough for reducing different environmental and camera parameters from the accuracy point of view. Different applications of DL methods have different goals. Since DL shows itself as the potential tool for all applications, DL techniques have to be robust against different types of environmental and camera impacts. Therefore, there is a need to introduce more effective DL-based distortion reduction techniques considering the purpose of applications so that they can accurately handle the application-specific images having many different environmental and camera impacts.
5. IoT network is vulnerable to cyber-attacks. Research shows DL models are also threatened by various types of attacks such as adversarial attacks (Ren et al. 2020), network attacks on DL architectures (Wu et al. 2020) and data poisoning attacks (Chou et al. 2018). Therefore, appropriate cyber-security protection mechanisms either need to be developed for integrating with or incorporated in IoT data gathering, transmission, and processing steps as well as DL training and testing phases.
6. For most of the applications, real datasets (covering all environmental impacts such as rain, fog, shadow, wind, darkness, and camera impacts such as lens blurriness, dirtiness, and barrel distortion) are not available. There exists a model that simulates the camera to generate the data for training traffic parking guidance systems considering the lens blur and lens distortion camera impacts (Tschentscher et al. 2017). The main loophole of this simulation model is that this uses only lens blur and lens distortion camera impacts. The advancement of DL techniques for the analysis and reduction of environmental and camera impacts require addressing the problem of the unavailability of the dataset mentioned before in this research challenge. Therefore, similar to the simulation model developed in Tschentscher et al. (2017), there is a need for the development of a simulation model which is capable of generating data that closely represents various real-world environmental deployment scenarios. The development of such a simulation model also demands a technique to validate the simulated data against their real-world counterparts.

## Conclusions

There is not a plethora of DL techniques available for analysing the impingement of environmental and camera lens distortions. From the literature, we perceive that DL-based image processing techniques can deal with uncertainties. These uncertainties are associated with images and their processing techniques that lead to non-deterministic problems. DL algorithms can handle these problems. This paper presents an SLR of the recent state-of-the-art DL-based approaches for analyzing and reducing environmental and camera lens impacts on images and presents some open research issues in the relevant areas. Even though DL techniques seem to be the potential to handle these impacts, still now there are many research gaps that need to be addressed in the future. Some of these research gaps are mentioned in Sect. 6. Moreover, there exist some techniques which alone cannot analyse and reduce the repercussions related to the impact of all environmental parameters. For instance, only one DL technique is not able to analyse all the environmental impacts such as rain, snow, fog, and many more while image capturing. The research study also shows sufficient work is not done to analyse the impact of wind on IoT images as compared to other factors such as rain, snow, and fog. We also acknowledge that image communication through IoT networks raises many research issues like less reliable data forwarding, low latency for real-time applications, and vulnerability to cyberattacks. This survey covers applications that use DL techniques to analyse and reduce camera impacts on IoT images. However, when sufficient DL-based techniques are available for such analysis and reduction in the future, the further survey can be conducted on specific applications such as autonomous driving.
